# EXAMINING THE RELATIONSHIP BETWEEN ALPHA-KLOTHO, INSULIN RESISTANCE, AND DIABETES IN OLDER ADULTS

**DOI:** 10.1093/geroni/igae098.2291

**Published:** 2024-12-31

**Authors:** Anam Ahmad, Michelle Shardell, Rita Kalyani, Richard Semba, Luigi Ferrucci

**Affiliations:** University of Maryland School of Medicine, Cary, North Carolina, United States; University of Maryland School of Medicine, Baltimore, Maryland, United States; Johns Hopkins University School of Medicine, Baltimore, Maryland, United States; Johns Hopkins University, Baltimore, Maryland, United States; National Institute on Aging, Baltimore, Maryland, United States

## Abstract

Higher circulating concentrations of alpha-klotho protein have been associated with greater longevity and inversely linked with aging-related phenotypes in human and animal studies. Impaired insulin signaling contributes to deregulated nutrient sensing, a hallmark of aging that plays a role in age-related adiposity, diabetes risk, and reduced lifespan. Whether alpha-klotho is part of this longevity pathway is unknown. We aimed to assess the relationships of alpha-klotho with insulin resistance and diabetes, and whether these relationships depended on adiposity in 860 adults aged ≥55 years from the Invecchiare in Chianti Study. Insulin resistance was operationalized using the homeostasis model for insulin resistance (HOMA-IR); diabetes was defined as having fasting blood glucose ≥126 mg/dL, glycosuria, taking diabetic medications, or a combination of patient self-report and diet assessment, and adiposity was defined using body mass index (BMI). Stratified linear and logistic regression were used to quantify associations of alpha-klotho with HOMA-IR and diabetes, respectively. Inverse probability weighting was used to address missing data. Among 394 overweight participants (BMI 25.0-29.9 kg/m2), each logarithm higher alpha-klotho (pg/mL) was associated with 0.42 units higher HOMA-IR (95% confidence interval [CI] 0.09-0.75) and 1.34 times higher, though not statistically significant, odds of diabetes (95% CI 0.35-5.10), after adjustment for covariates. There was little evidence of association among those with BMI< 25.0kg/m2 or BMI≥30 kg/m2. The positive association with HOMA-IR in overweight only and no significant association with diabetes may reflect an alternative hypothesis of alpha-klotho overexpression promoting insulin resistance. Further studies are needed to understand the mechanism of these findings.

